# Subcutaneous Abatacept vErsus Intravenous Abatacept: A Phase IIIb Noninferiority Study in Patients With an Inadequate Response to Methotrexate

**DOI:** 10.1002/art.30463

**Published:** 2011-10

**Authors:** M C Genovese, A Covarrubias, G Leon, E Mysler, M Keiserman, R Valente, P Nash, J A Simon-Campos, W Porawska, J Box, C Legerton, E Nasonov, P Durez, R Aranda, R Pappu, I Delaet, J Teng, R Alten

**Affiliations:** 1 Stanford UniversityPalo Alto, California; 2 Centro Medico De Las AmericasMerida, Mexico; 3 Instituto De Ginecologia Y ReproduccionLima, Peru; 4 Organización Médica de InvestigaciónBuenos Aires, Argentina; 5 Pontificial Catholic University School of MedicinePorto Alegre, Brazil; 6 Physician Research CollaborationLincoln, Nebraska; 7 University of QueenslandBrisbane, Queensland, Australia; 8 Centro De Especialidades Médicas/HRAEPYMerida, Mexico; 9 Poznanski Osrodek Medyczny NovamedPoznań, Poland; 10 Box Arthritis and Rheumatology of the CarolinasCharlotte, North Carolina; 11 Low Country RheumatologyCharleston, South Carolina; 12 Institute of RheumatologyMoscow, Russia; 13 Cliniques Universitaires Saint-Luc and Université Catholique de LouvainBrussels, Belgium; 14 Bristol-Myers SquibbPrinceton, New Jersey; 15 Schlosspark-Klinik, Teaching Hospital of the Charité University Medicine BerlinBerlin, Germany

## Abstract

**Objective:**

To compare the efficacy and safety of subcutaneous (SC) and intravenous (IV) abatacept.

**Methods:**

In this phase IIIb double-blind, double-dummy, 6-month study, patients with rheumatoid arthritis (RA) and inadequate responses to methotrexate were randomized to receive 125 mg SC abatacept on days 1 and 8 and weekly thereafter (plus an IV loading dose [∼10 mg/kg] on day 1) or IV abatacept (∼10 mg/kg) on days 1, 15, and 29 and every 4 weeks thereafter. The primary end point for determining the noninferiority of SC abatacept to IV abatacept was the proportion of patients in each group meeting the American College of Rheumatology 20% improvement criteria (achieving an ACR20 response) at month 6. Other efficacy end points, immunogenicity, and safety were also assessed.

**Results:**

Of 1,457 patients, 693 of 736 (94.2%) treated with SC abatacept and 676 of 721 (93.8%) treated with IV abatacept completed 6 months. At month 6, 76.0% (95% confidence interval 72.9, 79.2) of SC abatacept–treated patients versus 75.8% (95% confidence interval 72.6, 79.0) of IV abatacept–treated patients achieved an ACR20 response (estimated difference between groups 0.3% [95% confidence interval –4.2, 4.8]), confirming noninferiority of SC abatacept to IV abatacept. Onset and magnitude of ACR responses and disease activity and physical function improvements were comparable between the SC and IV abatacept–treated groups. The proportions of adverse events (AEs) and serious AEs over 6 months were 67.0% and 4.2%, respectively, in the SC abatacept–treated group and 65.2% and 4.9%, respectively, in the IV abatacept–treated group, with comparable frequencies of serious infections, malignancies, and autoimmune events between groups. SC injection site reactions (mostly mild) occurred in 19 SC abatacept (IV placebo)–treated patients (2.6%) and 18 IV abatacept (SC placebo)–treated patients (2.5%). Abatacept-induced antibodies occurred in 1.1% of SC abatacept–treated patients and 2.3% of IV abatacept–treated patients.

**Conclusion:**

SC abatacept provides efficacy and safety comparable with that of IV abatacept, with low immunogenicity and high retention rates, consistent with the established IV abatacept profile. Rates of injection site reactions were low. SC abatacept will provide additional treatment options, such as an alternative route of administration, for patients with RA.

The first biologic therapies were approved for the treatment of rheumatoid arthritis (RA) more than a decade ago (1); since then, a variety of agents with differing mechanisms of action have been approved. Many factors influence the selection of an appropriate RA therapy. Most importantly, safety and efficacy must be considered in the context of the patient's clinical profile; however, the route of administration of the agent can also be a determining factor.

The efficacy and safety of abatacept, a selective T cell costimulation modulator, have been established across a range of RA patient populations (2–9). Currently, abatacept is approved for monthly intravenous (IV) administration according to a weight-tiered dosing regimen in patients with moderate-to-severe RA (10). The availability of a subcutaneous (SC) formulation of abatacept would increase the treatment options available to patients with RA, particularly those wishing to self-administer their therapy.

An SC formulation of abatacept has been studied in multiple phase II and III trials. SC abatacept administered at a fixed dose of 125 mg/week was well tolerated over 3 months, with a safety and immunogenicity profile similar to that of the IV regimen (∼10 mg/kg monthly) (11,12). In the phase IIIb ACCOMPANY (Abatacept in Subjects with Rheumatoid Arthritis Administered Plus or Minus Background Methotrexate Subcutaneously) study, SC abatacept demonstrated acceptable tolerability with minimal injection site reactions and low rates of immunogenicity when administered as a monotherapy, or with background methotrexate (MTX), even in the absence of an IV loading dose (13,14). Improvements in disease activity were observed across all SC treatment groups (13,14). Here we report the outcome of the multinational, phase IIIb, noninferiority ACQUIRE (Abatacept Comparison of Subcutaneous versus Intravenous in Inadequate Responders to Methotrexate) study, which directly compared the efficacy and safety of SC abatacept with IV abatacept.

## PATIENTS AND METHODS

### Patient population

Patients who met the American College of Rheumatology (ACR) 1987 revised criteria for the classification of RA (15) who were in functional classes I, II, or III according to the ACR 1991 revised criteria (16) and who had active disease were eligible for inclusion. Patients had to have had an inadequate response to ≥3 months of MTX therapy (≥15 mg/week), with ≥10 swollen joints, ≥12 tender joints, and C-reactive protein (CRP) levels of ≥0.8 mg/dl at randomization. Patients were screened for tuberculosis (TB) at baseline and excluded if there was current clinical/radiographic/laboratory evidence of active TB or a history of active TB within the last 3 years, even if treated. Patients with a history of active TB >3 years earlier were included only with documentation of appropriate treatment. Patients with latent TB were included if treatment with isoniazid (9-month course) had been initiated at least 4 weeks prior to receiving study drug and if active infection was ruled out by negative chest radiographic findings at enrollment.

### Study design

This was a 6-month, multinational, phase IIIb, randomized, double-blind, double-dummy study (17), with an open-label long-term extension period (results to be presented separately). The protocol and patients' informed consent received Institutional Review Board/Independent Ethics Committee approval. The study was conducted in accordance with the Declaration of Helsinki and was consistent with the International Conference on Harmonization and Good Clinical Practice.

Patients were randomized (1:1), with stratification by body weight (<60 kg, 60–100 kg, >100 kg), to receive abatacept either by SC injections (125 mg) on days 1 and 8 and weekly thereafter or by IV infusions (∼10 mg/kg based on weight range) on days 1, 15, and 29 and every 4 weeks thereafter. Patients randomized to SC abatacept also received an IV abatacept loading dose (∼10 mg/kg based on weight range) on day 1, to ensure that the trough serum concentration of abatacept required to achieve full receptor occupancy, and thus maximal T cell inhibition (i.e., 10 μg/ml) (11,12,18), was achieved as quickly as possible.

A double-dummy design was used to maintain blinding. Patients randomized to the SC abatacept group received IV placebo on days 15 and 29 and every 4 weeks thereafter, and patients randomized to the IV abatacept group received SC placebo on day 8 and weekly thereafter. For all patients, SC injection was administered ∼30 minutes after the end of IV infusion. Patients and study site personnel remained blinded with regard to treatment assignments during the double-blind period.

Patients continued taking MTX at the same dosage they were receiving at randomization (minimum 15 mg/week), and changes were not permitted during the first 6 months (except if toxicity occurred). All other disease-modifying antirheumatic drugs were discontinued at least 4 weeks prior to study treatment (or 8 weeks for leflunomide). Low-dose oral corticosteroids (≤10 mg/day prednisone equivalent) were permitted throughout the trial; the dosage had to be stable for 25–28 days prior to study entry. A maximum of 2 of the following high-dose corticosteroid courses were permitted, as long as they were not within 28 days of the month 6 visit: a short (maximum 2 weeks) oral course of high-dose corticosteroids, a single intramuscular (IM) dose of corticosteroids, or a single intraarticular (IA) injection of corticosteroids (any joint that received an IA injection was counted as having “active” disease for the remainder of the study).

### Efficacy assessments

Efficacy assessments were performed on days 1, 15, and 29 and then every 4 weeks thereafter. The primary end point for determining the noninferiority of SC abatacept to IV abatacept was the proportion of patients in each group meeting the ACR 20% improvement criteria (achieving an ACR20 response) (19) at month 6. The proportions of patients achieving ACR50 and ACR70 responses at month 6 were secondary end points. Subgroup analyses (prespecified) are also presented for ACR responses according to patient weight range (<60 kg, 60–100 kg, >100 kg).

Physical function, measured using the patient-reported Health Assessment Questionnaire disability index (HAQ DI) (20), was a secondary end point. The Disease Activity Score in 28 joints (DAS28) (21) using the CRP level (DAS28-CRP) was a tertiary end point. Mean changes in HAQ DI score and DAS28-CRP at month 6 are summarized. The proportions of patients achieving a HAQ DI response (improvement of ≥0.3 units from baseline) (22), a low disease activity state (DAS28-CRP of ≤3.2), and DAS28-defined remission (DAS28-CRP of <2.6) are summarized. Additional patient-reported outcomes, such as pain and global assessment of disease activity (assessed using a 0–100-mm visual analog scale), are reported.

### Safety and immunogenicity assessments

Safety assessments were classified using the *Medical Dictionary for Regulatory Activities* (MedDRA). Patients were monitored for SC injection site reactions and for acute infusion reactions (within 1 hour of the start of IV infusion). Injection site and infusion reactions were prespecified, based on a list of MedDRA Preferred Terms. Autoimmune events were also prespecified based on a list of MedDRA Preferred Terms.

Blood samples for immunogenicity assessments were collected prior to abatacept administration on day 1 and at months 3 and 6. The primary analysis method for detection of antiabatacept and anti–cytotoxic T lymphocyte–associated protein 4 Tip (anti–CTLA-4-T; the abatacept molecule without the Ig portion) antibodies was a validated enzyme-linked immunosorbent assay. An electrochemiluminescence assay (Meso-Scale Discovery) was used as a secondary assay for the assessment of immunogenicity.

### Statistical analysis

The noninferiority margin for 70% preservation of the minimum effect of IV abatacept was 7.5% ([1 − 0.7] × 25%), based on a minimum expected ACR20 response benefit of IV abatacept 25% greater than that of placebo (7,23). If the lower boundary of the 2-sided 95% confidence interval (95% CI) of the difference between SC abatacept and IV abatacept is at least −7.5%, SC abatacept can be considered noninferior to IV abatacept. This allows for a maximum difference of –2.1% (95% CI –7.5, 3.2) between the ACR20 response to SC abatacept and that to IV abatacept. A sample size of 1,440 was calculated to provide ∼80% power to demonstrate that the lower limit of the 2-sided 95% CI of the difference in ACR20 response rates between SC abatacept and IV abatacept was at least –7.5%.

Efficacy data were assessed for both the per-protocol and the intent-to-treat (ITT) populations. The ITT population includes all randomized patients who received at least 1 dose of medication; the per-protocol population excludes patients with protocol violations. Results from 1 investigating site were excluded from efficacy analyses owing to noncompliance with good clinical practice (8 patients), but were included in all safety analyses. Based on regulatory guidance, the per-protocol population was the primary analysis set for the primary end point; ACR responses over time were analyzed for both the per-protocol and ITT populations. Data are presented for the ITT population unless stated otherwise. Safety data are presented for all patients who received at least 1 dose of study medication.

Baseline demographics and clinical characteristics were analyzed descriptively for all patients. Treatment differences were calculated for efficacy assessments, with 95% CIs. For ACR or HAQ DI responses, patients who discontinued were considered nonresponders. For mean change in HAQ DI, DAS28-CRP, and patient-reported outcomes, missing values were imputed using a last observation carried forward analysis (we excluded patients for whom only baseline observations were available). For low disease activity state or DAS28-defined remission, we included patients for whom data were available at the visit of interest (as-observed analysis). Changes in HAQ DI score and DAS28-CRP were assessed using analysis of covariance (which included treatment as the main factor and baseline values and weight stratification as covariates), adjusted mean ± SEM, and 95% CI for adjusted mean difference between treatment groups.

## RESULTS

### Patient disposition

**Figure 1:**
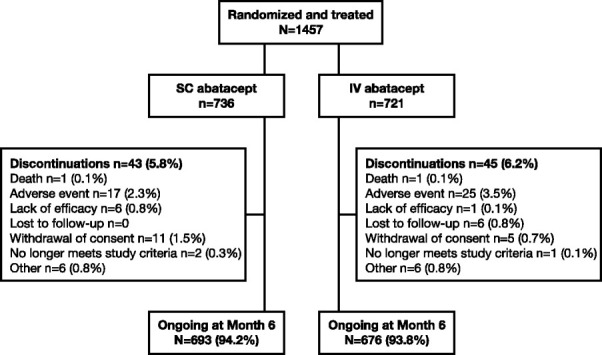
Patient disposition over 6 months (intent-to-treat population). SC = subcutaneous; IV = intravenous.

A total of 1,457 patients were randomized and treated with abatacept plus MTX; 736 were treated with SC abatacept plus IV placebo and 721 were treated with IV abatacept plus SC placebo (Figure 1). Of these randomized and treated patients, 40 (5.4%) in the SC abatacept–treated group and 38 (5.3%) in the IV abatacept–treated group had at least 1 relevant protocol deviation. The most frequent protocol deviations were joint count of <10 swollen joints or <12 tender joints at randomization, CRP level <0.5 mg/dl prior to or on day 1, or IA/IM/IV steroid injections or high oral steroid bursts (defined as oral steroid use >10 mg prednisone equivalent for more than 14 days) within 28 days prior to the month 6 assessment. Over 6 months, 43 SC abatacept–treated patients (5.8%) and 45 IV abatacept–treated patients (6.2%) discontinued the study; 693 SC abatacept–treated patients (94.2%) and 676 IV abatacept–treated patients (93.8%) were still participating in the study at month 6 (Figure 1).

### Baseline demographics and clinical characteristics

Baseline demographics and clinical characteristics were similar across the SC and IV abatacept–treated groups, and were comparable for per-protocol and ITT populations (Table 1). In the SC and IV abatacept–treated groups, the mean RA duration was 7.6 and 7.7 years, respectively, while the mean tender and swollen joint counts were 30.1 and 20.4, respectively, for the SC abatacept–treated group and 29.1 and 19.4, respectively, for the IV abatacept–treated group (ITT population) (Table 1).

**Table 1:** Baseline demographics and clinical characteristics, and permitted concomitant medications*

	Per-protocol population		ITT population	
		
	SC abatacept + MTX (n = 696)	IV abatacept + MTX (n = 683)	SC abatacept + MTX (n = 736)	IV abatacept + MTX (n = 721)
Age, years	49.9 ± 13.0	49.9 ± 12.7	49.9 ± 13.2	50.1 ± 12.6
Weight, kg	72.1 ± 18.1	71.5 ± 17.5	72.0 ± 18.0	71.8 ± 17.6
Weight group, no. (%)				
<60 kg	175 (25.1)	171 (25.0)	186 (25.3)	179 (24.8)
60–100 kg	464 (66.7)	465 (68.1)	492 (66.8)	489 (67.8)
>100 kg	57 (8.2)	47 (6.9)	58 (7.9)	53 (7.4)
Women, %	84.2	80.4	84.4	80.4
Caucasian, %	74.1	73.9	74.7	74.5
Disease duration, years	7.6 ± 8.0	7.7 ± 7.9	7.6 ± 8.1	7.7 ± 7.8
Tender joints	30.0 ± 14.1	29.2 ± 13.1	30.1 ± 14.1	29.1 ± 13.3
Swollen joints	20.5 ± 9.4	19.6 ± 8.5	20.4 ± 9.6	19.4 ± 8.6
HAQ DI score	1.7 ± 0.7	1.7 ± 0.7	1.7 ± 0.7	1.7 ± 0.7
Patient's assessment of pain, 0–100-mm VAS	68 ± 20.0	66.9 ± 20.5	67.8 ± 20.1	66.8 ± 20.5
Patient's global assessment of disease activity, 0–100-mm VAS	67.2 ± 20.1	65.2 ± 19.9	66.8 ± 20.4	64.9 ± 20.0
Physician's global assessment of disease activity, 0–100-mm VAS	64.3 ± 16.5	63.4 ± 16.3	64.3 ± 16.5	63.1 ± 16.6
CRP level, mg/dl	2.7 ± 2.9†	2.7 ± 2.9	2.6 ± 2.9‡	2.7 ± 2.9
DAS28-CRP	6.25 ± 0.84§	6.22 ± 0.83	6.23 ± 0.85¶	6.20 ± 0.84
Rheumatoid factor positive, no. (%)	582 (85.1)	583 (86.5)	614 (84.8)	611 (85.9)
MTX dose, mg/week	16.3 ± 3.6	16.5 ± 3.7	16.3 ± 3.6	16.5 ± 3.8
Biologic therapy prior to enrollment, no. (%)				
Biologics	24 (3.4)	31 (4.5)	32 (4.3)	43 (6.0)
Anti-TNF therapy	23 (3.3)	31 (4.5)	31 (4.2)	43 (6.0)
Etanercept	12 (1.7)	10 (1.5)	17 (2.3)	18 (2.5)
Adalimumab	5 (0.7)	11 (1.6)	9 (1.2)	14 (1.9)
Infliximab	6 (0.9)	10 (1.5)	11 (1.5)	17 (2.4)
Anakinra	1 (0.1)	0	1 (0.1)	2 (0.3)
Tocilizumab	0	1 (0.1)	0	1 (0.1)
Concomitant medication over the 6-month study period#				
Corticosteroids (oral and/or injectable), no. (%)	500 (71.8)	508 (74.4)	531 (72.1)	538 (74.6)
Oral corticosteroid dose, mg/day	4.7 ± 4.4	5.1 ± 7.0	4.8 ± 4.5	5.2 ± 6.9
High-dose corticosteroids, no. (%)				
>1	8 (1.1)	7 (1.0)	10 (1.4)	12 (1.7)
>2 IA injections	3 (0.4)	2 (0.3)	4 (0.5)	7 (1.0)

* Except where indicated otherwise, values are the mean ± SD. SC = subcutaneous; MTX = methotrexate; IV = intravenous; ITT = intent-to-treat; HAQ DI = Health Assessment Questionnaire disability index; VAS = visual analog scale; CRP = C-reactive protein; DAS28-CRP = Disease Activity Score in 28 joints using the CRP level; anti-TNF = anti–tumor necrosis factor; IA = intraarticular.

† n = 694.

‡ n = 734.

§ n = 693.

¶ n = 733.

# Includes data from up to 56 days after the last dose of study drug.

### Permitted concomitant medications

All patients received MTX, and the mean baseline dose was comparable between groups (Table 1). The proportion of patients receiving concomitant corticosteroids over 6 months was 72.1% in the SC abatacept–treated group and 74.6% in the IV abatacept–treated group (ITT population) (Table 1). High-dose corticosteroid use was infrequent and comparable between groups; 10 patients (1.4%) and 12 patients (1.7%) in the SC and IV abatacept–treated groups, respectively, received at least 1 high-dose oral, IM, or IV corticosteroid treatment (ITT population) (Table 1).

### Clinical efficacy

#### ACR responses

**Figure 2:**
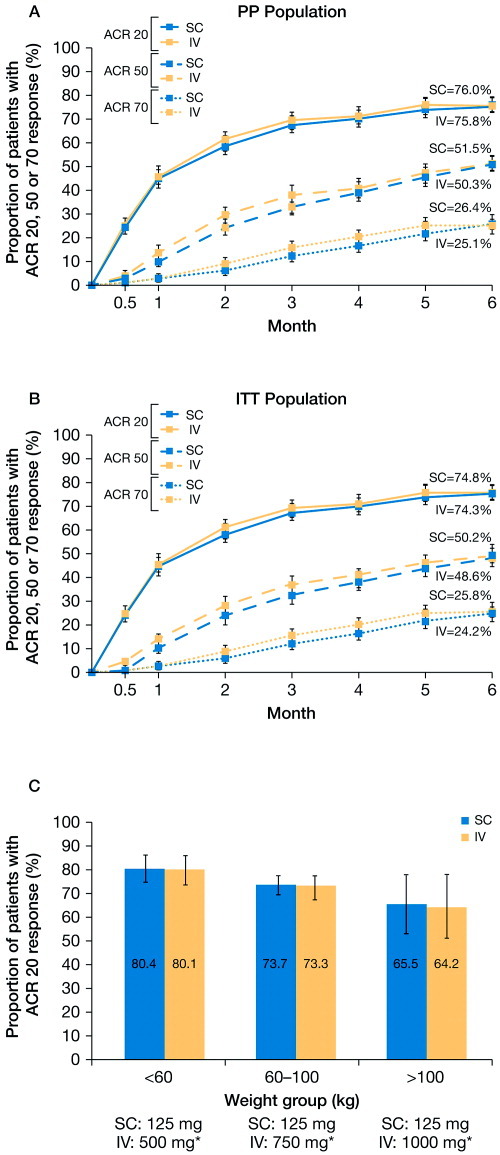
Proportions of SC or IV abatacept–treated patients meeting the American College of Rheumatology 20% improvement criteria (achieving an ACR20 response) over 6 months, as well as proportions of patients achieving ACR50 or ACR70 responses during the same time period. **A**, ACR20 (top), ACR50 (middle), and ACR70 (bottom) responses over 6 months for the per-protocol (PP) population (n = 693 in the SC abatacept–treated group, n = 678 in the IV abatacept–treated group). **B**, ACR20 (top), ACR50 (middle), and ACR70 (bottom) responses over 6 months for the intent-to-treat (ITT) population (n = 733 in the SC abatacept–treated group, n = 716 in the IV abatacept–treated group). **C**, ACR20 responses at month 6 by weight category for the ITT population (n = 733 in the SC abatacept–treated group, n = 716 in the IV abatacept–treated group). Not included are data on 8 patients who were excluded from all efficacy analyses owing to site noncompliance with study procedures. Asterisks indicate dosages corresponding to ∼10 mg/kg, according to weight range. Error bars represent 95% confidence intervals. See Figure 1 for other definitions.

The study met the primary objective of showing noninferiority of SC abatacept to IV abatacept. The proportion of patients achieving an ACR20 response at month 6 (primary end point; per-protocol population) was 76.0% (95% CI 72.9, 79.2) for the SC abatacept–treated group and 75.8% (95% CI 72.6, 79.0) for the IV abatacept–treated group (Figure 2A); the estimate of difference between groups was 0.3% (95% CI –4.2, 4.8). ACR50 and ACR70 response rates over 6 months were also comparable between the SC and IV abatacept–treated groups (per-protocol population) (Figure 2A). ACR20, ACR50, and ACR70 response rates over 6 months were comparable between the SC and IV abatacept–treated groups in the ITT population, and were similar to those in the per-protocol population (Figures 2A and B). The estimate of difference between the SC and IV abatacept–treated groups for the ACR20 response was 0.5% (95% CI –4.0, 4.9) (ITT population) (Figure 2B). ACR20, ACR50, and ACR70 response rates continued to increase over 6 months (Figure 2). When the primary end point (ACR20 response at month 6) was analyzed by weight, comparable results were observed between the SC and IV abatacept–treated groups (Figure 2C).

#### Physical function

**Figure 3:**
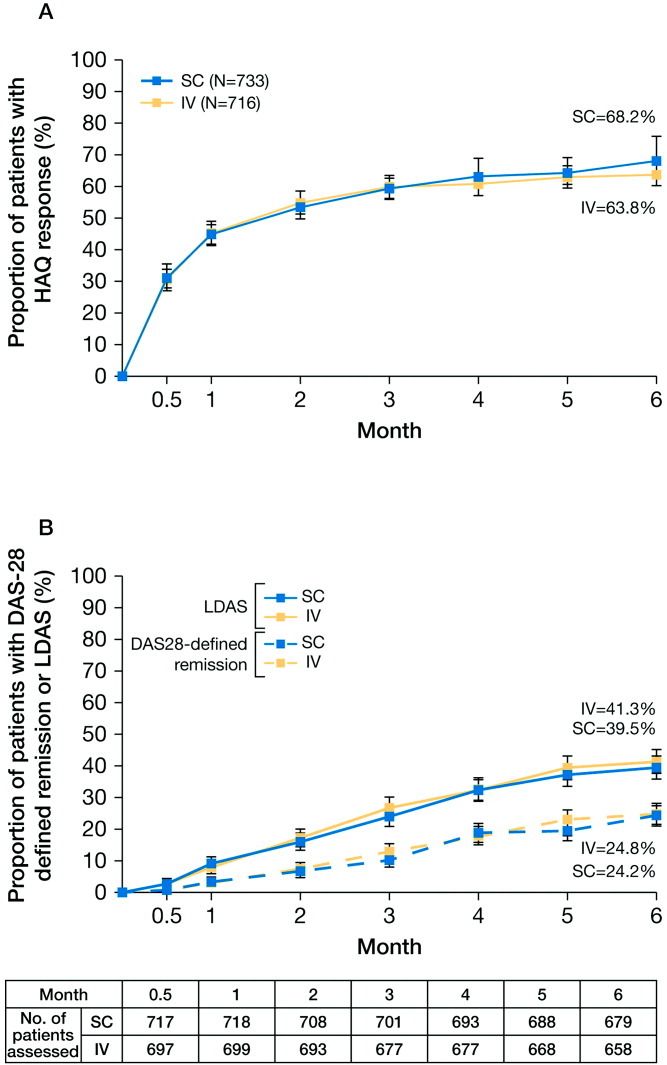
Functional disability and disease activity over 6 months in SC or IV abatacept–treated patients (intent-to-treat [ITT] population). **A**, Health Assessment Questionnaire (HAQ) disability index response (improvement of ≥0.3 units from baseline) over 6 months. **B**, Low disease activity state (LDAS) (Disease Activity Score in 28 joints using the C-reactive protein level [DAS28-CRP] of ≤3.2) (top) and DAS28-defined remission (DAS28-CRP of <2.6) (bottom) over 6 months (as-observed analysis). Not included are data on 8 patients who were excluded from all efficacy analyses owing to site noncompliance with study procedures. Error bars represent 95% confidence intervals. See Figure 1 for other definitions.

Improvements in HAQ DI score were comparable between the SC and IV abatacept–treated groups. The proportion of HAQ DI responders was 68.2% (95% CI 64.8, 71.6) for the SC abatacept–treated group and 63.8% (95% CI 60.3, 67.3) for the IV abatacept–treated group at month 6 (estimate of difference 4.5% [95% CI –0.4, 9.4]) (Figure 3A). The adjusted mean ± SEM change from baseline to month 6 in HAQ DI scores was –0.69 ± 0.02 and –0.70 ± 0.02 in the SC and IV abatacept–treated groups, respectively.

#### Disease activity

The mean ± SD baseline DAS28-CRP was similar in the SC and IV abatacept–treated groups (Table 1). At month 6, the mean ± SD DAS28-CRP was 3.7 ± 1.3 in both the SC and IV abatacept–treated groups, representing adjusted mean ± SEM improvements from baseline of –2.57 ± 0.05 and –2.55 ± 0.05, respectively.

At month 6, 39.5% (95% CI 35.8, 43.1) and 41.3% (95% CI 37.6, 45.1) of patients in the SC and IV abatacept–treated groups, respectively, had achieved a low disease activity state, and 24.2% (95% CI 20.9, 27.4) and 24.8% (95% CI 21.5, 28.1), respectively, had achieved DAS28-defined remission (Figure 3B). The estimates of treatment difference between the SC and IV abatacept–treated groups were −1.9% (95% CI −7.2, 3.4) and −0.7% (95% CI −5.3, 4.0) for low disease activity state and DAS28-defined remission, respectively.

#### Patient's assessment of pain and disease activity

At month 6, the adjusted mean ± SEM percent improvement from baseline in pain was 49.1 ± 1.74% and 44.9 ± 1.77% in the SC and IV abatacept–treated groups, respectively (adjusted difference from IV 4.2% [95% CI –0.7, 9.1]). For patient's global assessment of disease activity, the adjusted mean ± SEM percent improvement from baseline was 48.1 ± 1.66% and 47.4 ± 1.68% in the SC and IV abatacept–treated groups, respectively, at month 6 (adjusted difference from IV 0.7% [95% CI –3.9, 5.4]).

### Safety

The safety profile observed with SC abatacept was generally comparable to that with IV abatacept (Table 2). The proportions of adverse events (AEs) and serious AEs were 67.0% and 4.2%, respectively, in the SC abatacept–treated group and 65.2% and 4.9%, respectively, in the IV abatacept–treated group. The AEs reported in ≥5% of patients in either the SC or IV abatacept–treated group were headache, nasopharyngitis, upper respiratory tract infection, diarrhea, and nausea. Discontinuations due to serious AEs occurred in 1.1% of the SC abatacept–treated patients and in 1.9% of the IV abatacept–treated patients; these differences were primarily driven by infections. No patients discontinued due to serious infections in the SC abatacept–treated group, while 4 patients (0.6%) discontinued in the IV abatacept–treated group.

**Table 2:** Safety summary (ITT population)*

	SC abatacept + MTX (n = 736)	IV abatacept + MTX (n = 721)
Deaths	2 (0.3)	5 (0.7)
Serious AEs	31 (4.2)	35 (4.9)
Discontinued due to serious AEs	8 (1.1)	14 (1.9)
AEs	493 (67.0)	470 (65.2)
Discontinued due to AE	15 (2.0)	25 (3.5)
Infections	234 (31.8)	221 (30.7)
Serious infections	5 (0.7)	10 (1.4)
Malignancies	3 (0.4)	5 (0.7)
Autoimmune events	7 (1.0)	6 (0.8)
SC injection site reactions	19 (2.6)	18 (2.5)
Hematoma	4 (0.5)	4 (0.6)
Pruritus	6 (0.8)	1 (0.1)
Erythema	5 (0.7)	1 (0.1)
Pain	1 (0.1)	4 (0.6)
Papule	1 (0.1)	3 (0.4)
Reaction	1 (0.1)	3 (0.4)
Rash	2 (0.3)	1 (0.1)
Urticaria	0	2 (0.3)
Other†	4 (0.5)	0

* Values are the number (%) of events. Safety data are based on all patients who received at least 1 dose of abatacept. Given the double-dummy study design, patients in the SC abatacept–treated group received IV placebo and patients in the IV abatacept–treated group received SC placebo. AEs = adverse events (see Table 1 for other definitions).

† Individual SC injection site reactions not reported in more than 1 patient overall.

Two and 5 deaths were reported in the SC and IV abatacept–treated groups, respectively. In the SC abatacept–treated group, these resulted from staphylococcal septicemia in one patient and an unknown cause in another patient (who had a prior history of heavy smoking and frequent alcohol use). The 5 deaths in the IV abatacept–treated group resulted from subarachnoid hemorrhage, adenocarcinoma of the gall bladder with metastases, necrotizing pneumonia, bowel infarction, and multiple organ failure with septic shock and lung sepsis.

#### Infections

The frequency of infections was comparable between the SC and IV abatacept–treated groups, with most events being mild or moderate in intensity. The most frequently reported infections were nasopharyngitis (5.6% and 5.8%), upper respiratory tract infection (4.8% and 5.1%), bronchitis (3.4% and 4.0%), urinary tract infection (2.9% and 2.6%), and pharyngitis (2.6% and 1.8%) in the SC and IV abatacept–treated groups, respectively.

Serious infections occurred in 5 patients (0.7%) in the SC abatacept–treated group and 10 patients (1.4%) in the IV abatacept–treated group. The most frequently reported serious infections were pneumonia (1 patient [0.1%] in the SC abatacept–treated group and 3 patients [0.4%] in the IV abatacept–treated group), gastroenteritis (1 patient [0.1%] in each group), and urinary tract infection (2 patients [0.3%] in the IV abatacept–treated group); all other serious infections occurred in only 1 patient overall. There were no opportunistic infections, including TB, in either treatment group.

#### Malignancies

Malignancies occurred in 3 patients (0.4%) in the SC abatacept–treated group (basal cell carcinoma in 2 patients and B cell lymphoma in 1 patient) and 5 patients (0.7%) in the IV abatacept–treated group (basal cell carcinoma, cervix carcinoma [stage 0], colon neoplasm, metastatic gall bladder cancer, and squamous cell carcinoma of the skin in 1 patient each).

#### Autoimmune events

Prespecified autoimmune events were reported in ≤1% of patients in each treatment arm (7 patients [1.0%] and 6 patients [0.8%] in the SC and IV abatacept–treated groups, respectively); all were mild or moderate in intensity. The most frequently reported autoimmune event was psoriasis (2 patients [0.3%] in the SC abatacept–treated group and 4 patients [0.6%] in the IV abatacept–treated group). Other autoimmune events reported in the SC abatacept–treated group were erythema nodosum, episcleritis, uveitis, Raynaud's syndrome, and Sjögren's syndrome (in 1 patient each); other autoimmune events reported in the IV abatacept–treated group were hyperthyroidism and Crohn's disease (in 1 patient each). The patient with Crohn's disease subsequently discontinued from the study; no other autoimmune event resulted in discontinuation or interruption of treatment.

#### Infusion- and injection-related events

Prespecified SC injection site reactions were reported in 19 patients (2.6%) in the SC abatacept–treated group and in 18 patients (2.5%) in the IV abatacept (SC placebo)–treated group (Table 2). All SC injection site reactions were mild (89.2%) or moderate (10.8%) in intensity, and none resulted in withdrawal from the study. The most frequently reported events in the SC and IV abatacept–treated groups, respectively, were pruritis (6 patients [0.8%] and 1 patient [0.1%]), erythema (5 patients [0.7%] and 1 patient [0.1%]), and hematoma (4 patients each [0.5% and 0.6%, respectively]). Pain at the injection site was rare (1 patient [0.1%] in the SC abatacept–treated group and 4 patients [0.6%] in the IV abatacept [SC placebo]–treated group).

Acute infusional events (occurring within 1 hour of the start of the IV infusion) were reported in 20 patients (2.7%) in the SC abatacept (IV placebo)–treated group. The majority of patients (n = 12) experienced events on day 1, after the IV abatacept loading dose, with the remaining patients (n = 8) experiencing an event upon receiving IV placebo. Acute infusional events were reported in 16 patients (2.2%) in the IV abatacept–treated group. The types of acute infusional events were similar in the SC and IV abatacept–treated groups. Events were mostly mild or moderate in intensity, and the most common events were urticaria (3 patients [0.4%] and 2 patients [0.3%] in the SC and IV abatacept–treated groups, respectively), nausea (2 patients [0.3%] in each group), headache (2 patients [0.3%] in each group), and increased blood pressure (3 patients [0.4%] and 1 patient [0.1%] in the SC and IV abatacept–treated groups, respectively).

One patient (0.1%) in each group had an anaphylactic reaction on day 1. The patient in the SC abatacept–treated group was reported to develop grade II anaphylaxis (moderate in intensity) with grade III skin rashes, a cough, hypotension, and tachycardia, subsequent to receiving the IV abatacept loading dose. The patient in the IV abatacept–treated group experienced grade III anaphylaxis (severe in intensity) with pruritus, dizziness, nausea, diaphoresis, eyelid edema, and a rash on the face and thorax. For both patients, the events resolved on day 2, and no further doses of abatacept were administered.

### Immunogenicity

In total, 3 patients (0.4%) and 5 patients (0.7%) tested seropositive for antiabatacept antibodies in the SC and IV abatacept–treated groups, respectively (Table 3). A total of 5 patients (0.7%) and 11 patients (1.5%) tested seropositive for anti–CTLA-4-T antibodies in the SC and IV abatacept–treated groups, respectively (Table 3). The presence of a positive antibody seroconversion did not appear to affect the efficacy or safety of abatacept (data not shown).

**Table 3:** Immunogenicity rate (ITT population)*

	Antiabatacept	Anti–CTLA-4-T	Total
SC			
Treatment visit	3/707 (0.4)	2/716 (0.3)	5/716 (0.7)
Posttreatment visit†	0/26	3/28 (10.7)	3/28 (10.7)
Overall	3/714 (0.4)	5/725 (0.7)	8/725 (1.1)
IV			
Treatment visit	5/691 (0.7)	4/702 (0.6)	9/702 (1.3)
Posttreatment visit†	0/29	7/31 (22.6)	7/31 (22.6)
Overall	5/698 (0.7)	11/710 (1.5)	16/710 (2.3)

* Values are the number/total number (%) of patients. Data are based on patients for whom immunogenicity assessments were available. Anti–CTLA-4-T = anti–cytotoxic T lymphocyte–associated protein 4 Tip (see Table 1 for other definitions).

† Assessed for up to 85 days after withdrawal from the study.

## DISCUSSION

A variety of biologic agents that are administered either via the SC route or via the IV route are now available for the treatment of RA. The T cell costimulation modulator, abatacept, is approved for IV administration, and the long-term efficacy and safety profile of IV abatacept in patients with an inadequate response to MTX is well established (8,9,24). The availability of an alternative formulation of abatacept for administration via the SC route would increase the treatment options for patients with RA.

To investigate the clinical profile of an SC formulation of abatacept, a noninferiority study was conducted in patients with moderate-to-severe RA with inadequate MTX response. The aim of this study was to demonstrate whether the efficacy of SC abatacept is comparable with that of IV abatacept. Based on guidelines and feedback from regulatory authorities (25–27), assessment of noninferiority of SC abatacept to IV abatacept can be demonstrated by a 70% preservation of the treatment effect (measured by the ACR20 response). This predefined stringent noninferiority margin translates into an allowable absolute maximum difference of –2.1% between SC abatacept and IV abatacept. Based on regulatory statistical guidance (26), a per-protocol population was used for the primary end point. This population is considered more conservative for study in noninferiority trials, since it excludes patients with protocol violations and is therefore more likely to reflect differences between treatment arms (28).

Results presented here demonstrate an estimate of difference of 0.3% between treatment groups in the per-protocol population, confirming noninferiority of SC abatacept to IV abatacept. Approximately three-fourths of patients achieved an ACR20 response by month 6 in both groups. The rate and magnitude of response were comparable between SC abatacept and IV abatacept, demonstrated by ACR responses and improvements in physical function, disease activity, and patient-assessed outcomes. These efficacy benefits confirm those from the phase IIIb ACCOMPANY study of SC abatacept (13,14).

The SC formulation of abatacept is administered as a fixed dose (125 mg) across all weight ranges. This approach has previously been investigated in a phase II trial, which demonstrated that, across a range of weight groups, SC fixed dosing of 125 mg/week (plus an IV loading dose of ∼10 mg/kg on day 1) resulted in median trough serum concentrations comparable to or higher than those observed with the approved IV weight-tiered dosing regimen (11,12) and above the minimum concentration predicted to exert maximal T cell inhibition (29). Importantly, in the current trial, CIs for ACR20 responses overlap between the weight categories in the SC abatacept–treated group, indicating that efficacy benefits are generally comparable across weight ranges. ACR20 response rates were comparable between the SC and IV abatacept–treated groups within each weight category, although rates in both treatment groups were numerically lower in the >100 kg weight group than in the other 2 groups (<60 kg and 60–100 kg).

The safety observed with SC abatacept in this 6-month study was consistent with that found in previous phase IIIb SC abatacept studies (13,14) and studies of long-term IV administration. No new clinically significant AEs were observed, and safety was generally comparable to that of IV abatacept, for which there are now >12,000 cumulative patient-years of exposure in 4,419 patients (30). Similar frequencies of AEs, serious AEs, infections, malignancies, and autoimmune events were reported in the SC and IV abatacept–treated groups. Discontinuations due to serious AEs were slightly less frequent in the SC abatacept–treated group than in the IV abatacept–treated group, primarily driven by a lower number of infections in the former group. Serious infections were reported in 5 patients (0.7%) in the SC abatacept–treated group and 10 patients (1.4%) in the IV abatacept–treated group. The proportion of serious infections with IV abatacept was comparable to previously reported rates in a similar patient population (24), while the proportion of serious infections with SC abatacept was toward the lower end of previous observations. Furthermore, no opportunistic infections, including TB, were reported during the treatment period; this is consistent with the overall low rates reported in multiple phase III studies of IV abatacept in a similar population (6,7,24). A high proportion of patients completed the study, and numbers were comparable between treatment arms. The proportion of patients who discontinued due to AEs was low, and <1% in each group discontinued due to lack of efficacy.

Important considerations with SC administration of biologic agents are injection site reactions and tolerability. SC drug administration can be associated with adverse reactions at the site of injection, particularly injection site pain (31,32). In this study the overall incidence of injection site reactions with SC abatacept was low (<3%) and consistent with previous reports (11–14), indicating that the SC formulation is not associated with significant AEs at the injection site. This is further supported by the demonstration that the injection site reactions were comparable between patients receiving SC abatacept and those receiving SC placebo in the IV abatacept–treated group, with injection site pain reported in 1 and 4 patients, respectively. The incidence of injection site reactions observed with SC administration of etanercept plus MTX has been reported to range from 6.5% over 3 months to 10% over 1 year (33–35), and rates with adalimumab plus MTX in the range of 19.5–26.1% over 1 year have been observed (31,36).

Another potential challenge associated with SC protein therapeutics is the development of immunogenicity, which can result in a diminished clinical response in patients with RA (37,38). In this 6-month study of patients with an inadequate response to MTX, the proportion of patients with abatacept-induced antibodies was ∼1% in the SC abatacept–treated group and ∼2% in the IV abatacept–treated group. This low rate of immunogenicity is consistent with that reported in other SC abatacept clinical trials, in patients with up to 18 months of exposure to abatacept, administered as monotherapy or with concomitant MTX (11–14). The results are also consistent with reported immunogenicity rates from clinical trials using IV abatacept (5,9,39). Importantly, no effect on efficacy or safety was observed in the few patients who developed antibody responses to abatacept.

Interpretation of these results should take into consideration the limitations of the study, and the safety results should be interpreted in the context of the 6-month study duration. Approximately 5% of patients in each treatment group had at least 1 protocol deviation, mostly related to disease activity at entry or concomitant steroid use. However, comparable efficacy benefits were observed regardless of whether the population was analyzed on a per-protocol or ITT basis, suggesting that the results of this study are robust. In addition, although patients were permitted up to 2 courses of high-dose corticosteroids, the proportions of patients receiving these were low and comparable between treatment groups, so this was not likely to have affected study outcomes.

Due to the nature of the trial, all participants received active treatment; it is possible that this could affect the study findings by introducing an element of patient or assessor bias. However, any effect would apply across both treatment arms and would therefore be unlikely to affect comparisons between SC and IV abatacept–treated groups.

In summary, SC abatacept demonstrates efficacy and safety that are comparable and consistent with the established IV abatacept profile, including low immunogenicity and high retention rates. Importantly, injection site reactions with the SC formulation were infrequent and mild. These data support the use of abatacept for SC administration, providing an additional and beneficial treatment option for patients with RA.
